# Management of the injured bowel: preserving bowel continuity as a gold standard

**DOI:** 10.1186/s12893-021-01332-x

**Published:** 2021-09-08

**Authors:** Camille Tantardini, Gaëlle Godiris-Petit, Séverine Noullet, Mathieu Raux, Fabrice Menegaux, Nathalie Chereau

**Affiliations:** 1 Department of Digestive and Endocrine Surgery, Pitié-Salpêtrière Hospital, Assistance Publique-Hôpitaux de Paris, Sorbonne Université, Paris, France; 2 Department of Anesthesiology and Critical Care, Pitié-Salpêtrière Hospital, Assistance Publique-Hôpitaux de Paris, Sorbonne Université, Paris, France

**Keywords:** Colon injury, Small bowel injury, Abdominal trauma, Fistula, Stoma

## Abstract

**Background:**

Management of bowel traumatic injuries is a challenge. Although anastomotic or suture leak remains a feared complication, preserving bowel continuity is increasingly the preferred strategy. The aim of this study was to evaluate the outcomes of such a strategy.

**Methods:**

All included patients underwent surgery for bowel traumatic injuries at a high volume trauma center between 2007 and 2017. Postoperative course was analyzed for abdominal complications, morbidity and mortality.

**Results:**

Among 133 patients, 78% had small bowel injuries and 47% had colon injuries. 87% of small bowel injuries and 81% of colon injuries were treated with primary repair or anastomosis, with no difference in treatment according to injury site (p = 0.381). Mortality was 8%. Severe overall morbidity was 32%, and abdominal complications occurred in 32% of patients. Risk factors for severe overall morbidity were stoma creation (p = 0.036), heavy vascular expansion (p = 0.005) and a long delay before surgery (p = 0.023). Fistula rate was 2.2%; all leaks occurred after repairing small bowel wounds.

**Conclusion:**

Primary repair of bowel injuries should be the preferred option in trauma patient, regardless of the site—small bowel or colon—of the injury. Stoma creation is an important factor for postoperative morbidity, which should be weighed against the risk of an intestinal suture or anastomosis.

## Background

Operative management of traumatic hollow viscus injuries has been a subject of much debate, and colon injury especially remains a feared entity [1, 2]. Over the three decades following World War II, colostomy creation was the standard treatment for traumatic colon injuries [2–5]. Civilian surgeons started attempting primary repairs and anastomoses at the end of the 1970s; this change was soon validated by dozens of articles, including five randomized controlled trials [6–10] and a meta-analysis [11].

However, close analysis of these studies shows that surgeons were still wary of primary repairs for the most severe digestive wounds [12–15]. Recent articles written by military surgeons dampen the enthusiasm for primary repairs of the colon, evoking high mortality rates in case of fistula [16]. In everyday practice, the inconvenience of carrying a stoma appears negligible next to the complications of an anastomosis leak [17].

In the era of damage-control laparotomy, the problem of bowel continuity might be seen as a secondary issue [18, 19]. Nevertheless, damage-control laparotomy should not be considered as routine management for all patients with abdominal trauma [20]. Definitive surgery should be conducted whenever possible—and thus, the question of interrupting bowel continuity remains a key issue.

Preservation of bowel continuity is the preferred strategy in our institution regardless of the site—small bowel or colon—of the injury. The aim of the study was to analyze our management of patients with traumatic bowel injuries, using a prospective patient registry in a high volume trauma center. We aimed to identify which factors influenced the decision to preserve or not preserve bowel continuity and which factors were predictors for morbidity and mortality.

## Material and methods

We reviewed a prospectively held database of consecutive patients admitted for abdominal trauma with lesions of the digestive tract from 1997 to 2017. Included patients had emergent laparotomy, during which injuries to the small bowel and/or the colon were confirmed and treated. Simple serosal tears and lesions to the mesentery with no repercussions on bowel vitality were excluded.

Demographic data, type of bowel injury, type of trauma, time to surgery, hemodynamic status, transfusion, and biologic and radiologic data were gathered from medical charts.

The overall severity of the trauma was evaluated in two ways: hemodynamic status upon admission (patients with a systolic blood pressure lower than 90 mmHg or receiving vasopressors were considered to be unstable), and severity scores such as the Injury Severity Score (ISS) and the New Injury Severity Score (NISS). Both scores rely on a thorough examination of the patient and an exhaustive list of all lesions found, and are scaled from 1 to 75. The most commonly used threshold to define severe trauma is 15. While the ISS was developed to predict mortality, the NISS is supposed to better predict in-hospital morbidity.

During surgery, four types of injury management were identified: simple suture, resection and anastomosis, bowel resection with the ends left stapled in the abdomen, and stoma (with or without prior resection). As previously stated, we aimed to preserve bowel continuity as often as possible. Simple suture, with interrupted or running stitches of absorbable thread, was preferred in case of small wounds with clean edges. Resection and anastomosis were used if the bowel was ischemic or containing several wounds on a small segment. Anastomosis without resection was used to treat transfixing perforations without ischemia [21]. Both types of anastomosis were analyzed as a single group. All anastomoses were carried out with staplers.

If preservation of bowel continuity was considered unadvisable, a stoma was made, either as a loop stoma or as a resection and double stoma. In unstable patients presenting with the “hypothermia, acidosis and coagulopathy” triad, damage control laparotomy (DCL) was performed; the bowel was stapled shut and left in the abdomen, with simple skin closure. If the patient survived, a second procedure was performed 24 to 48 h later for definitive treatment of the bowel injury.

Postoperative course was analyzed for complications. Specific morbidity included intra-abdominal abscess, anastomotic or suture leak, wound abscess, and renewed intra-abdominal bleeding. Morbidity was defined as complications occurring during the hospital stay or in the month following surgery. The severity of complications was evaluated with the Clavien–Dindo classification. Mortality was defined as in-hospital death.

Chi-squared tests, Fisher’s exact tests and Student’s t tests were used for univariate analyses. Factors identified with an alpha level less than or equal to 0.05 were tested using logistic regression. Results are presented as means ± SD, as medians and IQRs, or percentages. A *p* value less than or equal to 0.05 was considered statistically significant. All tests were two tailed. Statistical analyses were conducted using SPSS (IBM Corp, SPSS Statistics for Windows, Version 23.0, Armonk, NY, USA).

## Results

Between 1997 and 2017, 165 patients were admitted for traumatic bowel injuries. Two patients died during surgery, 8 patients had lesions of the mesentery that did not compromise the intestinal tissue, 17 had only serosal wounds of minimal severity, and 5 patients had charts that were too incomplete to be included. A total of 133 patients (81% male) with a median age of 33.4 (i.q.r 11–79) years were included.

Fifty-three patients (40%) were admitted for blunt traumas, 54 (40%) were admitted for stab wounds, and 26 (20%) were admitted for gunshot wounds. Most patients (n = 93, 70%) had at least one injured organ other than the small bowel or colon. Thirty-eight patients (29%) were in shock on admission. Fifty-five patients (41%) needed a blood transfusion during the first 24 h, including 26 patients who required 6 red blood cell (RBC) units or more. (Table 1).

**Table 1:** Data on admission

	n (%)
Patients	
Male sex	108 (81%)
Median age (years)	33.4 [11–79]
Type of trauma	
Blunt	53 (40%)
Stab wounds	54 (40%)
Gunshot wounds	26 (20%)
Severity of trauma	
Median ISS	17 [4–75]
Median NISS	25 [9–75]
Delay to surgery	
Median time (h)	3 [1–96]
Patients over the 6-h limit	30 (23%)
Bowel injuries	
Small bowel injuries	104 (78%)
Colon injuries	62 (47%)
Associated injuries	
None	40 (30%)
Thorax	45 (35%)
Limb or pelvic bones	43 (32%)
Abdominal solid viscus (liver, spleen, pancreas)	34 (26%)
Large blood vessels	26 (20%)
Spine injury	25 (19%)
Urinary tract	25 (19%)
Other abdominal hollow viscus	14 (10%)
Stomach	11
Duodenum	1
Rectum/anus	2
Face	13 (10%)
Central Nervous System	12 (9%)
Hemodynamic status on admission	
Shock (SAP < 90 mmHg and/or need for vasopressor use)	38 (29%)
Vascular expansion (mL, mean and SD)	5091 (SD 3025)
Transfusion (RBC units, mean and SD)	4 (SD 7.5)
Polytransfusion (over 6 RBC units in the first 24 h)	26 (20%)

*ISS*  injury severity score, *NISS*  new injury severity score, *SAP*  systolic arterial pressure, *RBC*  red blood cell

One hundred and four patients (78%) had small bowel injuries, and 62 patients (47%) had colon injuries. Most patients had a primary repair or a resection and anastomosis (Table 2). There was no significant difference between the choice of treatment for small bowel injuries and that for colon injuries (p = 0.318). Concerning the patients themselves, 71 (53%) had only small bowel injuries, 29 (22%) had only colon injuries, and 33 (25%) had injuries in both sites. There was no significant difference in terms of preservation of bowel continuity between the three groups (p = 0.333).

**Table 2:** Treatment of intestinal injuries

	Small bowel injuries	Colon injuries	p
Total injuries	107	64	
Primary repair	28	16	0.866
Anastomosis	65	36	0.308
Ileo-ileal	65	0	
Ileo-colonic	0	14	
Colo-colonic	0	19	
Colorectal	0	3	
Resection and stoma	8	9	0.164
Lateral stoma	4	1	0.652
Resection during DCL	2	2	0.631
Total–preserved continuity (%)	93 (87%)	52 (81%)	0.318

*DLC*  damage control laparotomy

Seventy-five patients (56%) had a single resection (with or without anastomosis) and/or repair, while 58 patients (44%) had several resections and/or repairs. There was no difference between these two groups in terms of preservation of bowel continuity (p = 0.348), morbidity (p = 0.401) or mortality (p = 0.445).

Mortality was 8% (n = 11), including 8 patients who died before the third postoperative day (POD) from uncontrollable bleeding due to severe disseminated intravascular coagulopathy (DIC). The remaining 3 patients died of septic shock.

On univariate analysis, the following factors had a pejorative impact on mortality: blunt trauma (p = 0.036), transfusion of at least 6 blood units (p = 0.001), ISS over 15 (p = 0.003), elevated NISS (p < 0.0001), lesion of the mesentery (p = 0.003), lesion of abdominal solid viscus (p = 0.032), and hemodynamic shock (p = 0.013).

Forty-three patients (32%) had abdominal complications. Among them, 3 patients had suture or anastomosis leakage, accounting for a fistula rate of 2.2%. Ten other patients had intra-abdominal sepsis unrelated to the management of their bowel injuries: 4 infected hematomas, 1 infected pancreatic necrosis, 1 rectal fistula, 1 collection in an old drainage site, and 3 peritonites. These complications and their management are detailed in Table 3.

**Table 3:** Abdominal complications and their consequences

Abdominal Complications	n (patients)	Emergent reoperation	Death
Wound infection	9	0	0
Hemorrhage	9	3	8
Intra-abdominal sepsis	10	5	1
Suture/anastomosis leakage	3	3	1
Intestinal obstruction	4	2	0
Fascial dehiscence	2	1	0
Others	97	4	0
Total (patients)	43	18	10

Concerning stoma creation, there were 15 ileostomies and 9 colostomies. Median delay for stoma reversal was 69 days for ileostomies, and 164 days for colostomies. Six patients had early ileostomy reversal, before POD 12. Five were successful, one patient had emergent laparotomy for anastomosis leakage and double-barrel ileostomy was created anew. He had uneventful ileostomy reversal 2 months later.

All three patients with anastomosis or suture leakage were initially admitted for small bowel injuries.

The first patient presented with bowel ischemia following mesenteric disinsertion; he had small bowel resection and ileoileal anastomosis. He suffered anastomosis leakage and peritonitis on POD 8, and was treated by emergent laparotomy and double-barrel ileostomy. He died a few days later from septic shock due to perineal gangrene.

The second patient had right colectomy and ileocolonic anastomosis, and small bowel resection with ileoileal anastomosis. On POD 1 he had emergent laparotomy for abdominal compartment syndrome. The small bowel anastomosis was ischemic; it was resected and made into a double-barrel ileostomy. The patient recovered and had ileostomy reversal six months later.

The last patient had four small bowel injuries and massive fecal contamination. He had two primary repairs and one resection and anastomosis, while the most proximal injury was made into a diverting stoma. Despite this precaution, he had emergent laparotomy on POD 14 for suture leakage; the segment of bowel with the failed repairs was resected and a stoma was made. However, he developed septic shock and died the following day.

Severe overall morbidity (grade 3 to 5 of the Clavier-Dindo classification) was 32% (n = 43), mostly correlated with intensive care complications (e.g., pulmonary and catheter infections, deep vein thrombosis, and anticoagulation accidents).

On multivariate analysis, risk factors for severe overall morbidity were the creation of a stoma (p = 0.036), heavy vascular expansion in the first 24 h (p = 0.005) and a long delay before surgery (p = 0.023). Risk factors for abdominal complications were heavy vascular expansion (p = 0.024) and transfusion (p = 0.048) in the first 24 h. Details of the univariate analyses are shown in Table 4.

**Table 4:** Univariate analyses for abdominal complications and severe morbidity

	Abdominal complications	No abdominal complications	p	Severe morbidity (Clavien–Dindo 3–5)	No to mild morbidity	p
Male	35	73	0.969	35	73	0.969
Female	8	17		8	17	
Blunt trauma	20	33	0.278	24	29	*0.009*
Open trauma	23	57		19	61	
Delay ≥ 6 h	10	20	0.59	10	20	0.238
Delay < 6 h	19	49		15	53	
Transfusion ≥ 6 RBC units	15	11	0.002*	19	7	< *0.001*
Transfusion < 6 RBC units	28	79		24	83	
Fecal contamination	9	11	0.189	10	10	0.067
No fecal contamination	34	79		33	80	
Associated injury	34	59	0.112	38	55	*0.001*
No associated injury	9	31		5	35	
Mesenteric injury	15	25	0.403	18	22	*0.041*
No mesenteric injury	28	65		25	68	
Other abdominal injury	17	17	*0.011*	18	16	*0.003*
No other abdominal injury	26	73		25	74	
Hemodynamic shock	17	21	0.053	24	14	< *0.001*
Hemodynamic stability	26	69		19	76	
Treatment of other abdominal/thoracic injury	22	26	*0.012*	24	24	*0.001*
No other injury treated	21	64		19	66	
ISS ≥ 15	26	51	0.678	35	42	< *0.001*
ISS < 15	17	39		8	48	
Stoma	14	11	*0.005*	15	10	0.001*
No stoma	29	79		28	80	
Mean age (years)	37.1	36.6	0.846	38.3	36.1	0.419
Mean delay before surgery (hours)	14	8.3	0.165	18.5	7	0.006*
Mean transfusion	10.3	5.4	< *0.001*	10.1	1.7	< *0.001*
(RBC units)						
Mean vascular expansion (mL)	3523	2664	0.008*	7088	4552	0.002*
Mean duration of surgery (hours)	3.15	2.65	0.175	3.5	2.5	*0.008*
Mean ISS	19.9	18.7	0.571	23.8	16.9	< *0.001*
Mean NISS	28.9	24.9	0.109	32.7	23	< *0.001*
Mean abdominal AIS	3.44	3.29	0.143	3.58	3.22	< *0.001*

Statistically significant results appear in italic

*Results marked with asterisks remained significant after logistic regression

Risk factors for the creation of a stoma were fecal contamination and polytransfusion (p < 0.001).

## Discussion

Bowel injuries are most common in abdominal trauma, and most general surgeons will be confronted to the problem of repairing these injuries [22]. Stoma creation has become less and less popular when dealing with traumatic bowel injuries. Stone and Fabian have compared stoma creation and primary repair for colon injuries in 139 patients, in a pioneer randomized study in 1979 [6]. Their conclusion was that primary repair was at least as safe as colostomy, in the absence of major risk factors such as arterial hypotension, delayed operation, multiple associated injuries, and destructive colon injuries requiring resection. Their study was followed by four other trials, all in favor of primary repair or anastomosis [7–10].

The notion of major risk factors, defining “high-risk patients”, is found in many studies that were published after Stone and Fabian’s. Even though most of these studies proclaim the superiority of primary repair, several authors recommend caution when high-risk patients are concerned and do not reject colostomy creation out of hand. Miller et al. used these major risk factors to define an algorithm for the management of colon injuries [13–15]. Following this algorithm, nondestructive colon injuries should be treated with primary repair, and destructive colon injuries should be treated with resection with anastomosis, provided the patient has no comorbidity and has received fewer than 6 RBC units. If these conditions are not met, the patient should be treated with fecal diversion. This algorithm was tested for 15 years in the same center. Ultimately, Sharpe et al. reported an 80.4% rate of primary repair or anastomosis, with a 2.5% rate of postoperative fistulas [15].

In our data, the factors that most influenced the surgeon’s decision were fecal contamination and the transfusion of at least 6 RBC units in the first 24 h. Ultimately, we had an 87% rate of primary repair or anastomosis for small bowel injuries and an 81% rate for colon injuries. In our experience, fistula rate was a low 2.2%, with no leakage after colon injury repair.

In the management of bowel injuries, it is usually understood that fecal diversion aims to protect high-risk patients from postoperative complications. However, in our study, a major risk factor for severe morbidity was the creation of a stoma; this was tested in a multivariate analysis against other factors such as trauma severity, shock on admission and polytransfusion. Sasaki et al. noted an increase in septic complications in the colostomy group in 1995, although some complications occurred at the time of stoma reversal [10]. In our series, seven patients with ileostomies had complications related to high output, and six had early ileostomy reversal (before the 12th POD), either to avoid these systemic complications or to counter them. Four patients with colostomies had wound infections.

As previously said, one of the most compelling reasons for making a stoma was fecal contamination; yet an increasing number of studies show that, contrary to polytransfusion, fecal contamination is not a risk factor for post-operative complications and should not be a contraindication to primary repair [23].

From our results, we consider fecal diversion to be useless in preventing severe complications. However, should an ileostomy be created at the time of emergent laparotomy, early stoma reversal may be considered to avoid further morbidity—such as short bowel syndrome—in stabilized patients [24, 25].

In 2019, the Eastern Association for the Surgery of Trauma released new recommendations for the management of colon injuries [26]. Unsurprisingly, following the trend of recent years, it is recommended that resection and anastomosis or primary repair be performed for low-risk patients, rather than colostomy. High-risk patients were defined as patients presenting with a delay longer than 12 h, hemodynamic shock, associated injuries, contamination, transfusion of over 6 RBC units, or left-sided colon injuries; for these patients, anastomosis or primary repair is conditionally recommended. Colostomy may still be used for selected patients.

Our findings are in accordance with these recommendations. Moreover, we found that the treatment of colon injuries should not be different from the treatment of small bowel injuries and that the site of injury did not influence either the method of repair or the postoperative outcome. Undergoing researches might lead to the discovery of performant sealants that, when applied on an intestinal suture, may prevent anastomotic leakages [27]; thus, the use of stoma in dealing with abdominal trauma might be further minimized.

## Conclusion

Bowel continuity should be preserved as often as possible when managing intestinal trauma, regardless of the site of injury. Anastomoses and sutures are safe in most cases, with a 2.2% fistula rate. Stoma creation is an important factor for postoperative morbidity, which should be weighed against the hypothetical risk of anastomosis leakage.

## Data Availability

The dataset generated and analyzed during the current study is not publicly available. Even though it is anonymized, the information contained may lead to a breach in patient privacy, as the circumstances of some patients’ injuries were detailed in the press (the terrorist attack in November 2013 being one example). It is available from the corresponding author on reasonable request.

## Abbreviations

ISS: Injury severity score
NISS: New injury severity score
DCL: Damage control laparotomy
RBC: Red blood cell
POD: Post-operative day
DIC: Disseminated intravascular coagulopathy
